# Dialysate interleukin-6 predicts increasing peritoneal solute transport rate in incident peritoneal dialysis patients

**DOI:** 10.1186/1471-2369-15-8

**Published:** 2014-01-10

**Authors:** Yeoungjee Cho, David W Johnson, David A Vesey, Carmel M Hawley, Elaine M Pascoe, Margaret Clarke, Nicholas Topley

**Affiliations:** 1 Department of Renal Medicine, Princess Alexandra Hospital, Brisbane, Australia; 2 School of Medicine, University of Queensland, Brisbane, Australia; 3 Translational Research Institute, University of Queensland, Brisbane, Australia; 4 Fresenius Medical Care, Sydney, Australia; 5 Institute of Translation, Innovation, Methodology and Engagement, Cardiff University School of Medicine, Cardiff, UK

**Keywords:** Biocompatible, Glucose degradation products, Interleukin-6, Peritoneal dialysis, Peritoneal solute transport rate, Peritonitis

## Abstract

**Background:**

Repeated exposure to peritoneal dialysis (PD) solutions contributes to cumulative intraperitoneal inflammation and peritoneal injury. The present study aimed to explore the capacity of dialysate interleukin-6(IL-6) to a) predict peritoneal membrane function and peritonitis in incident PD patients, and b) to evaluate the influence of neutral pH, low glucose degradation product (GDP) PD solution on dialysate IL-6 levels.

**Methods:**

The study included 88 incident participants from the balANZ trial who had completed 24-months of follow-up. Change in peritoneal solute transport rate (PSTR) and peritonitis were primary outcome measures, and the utility of IL-6 and IL-6 appearance rate (IL-6 AR) in predicting these outcomes was analyzed using multilevel linear regression and Cox proportional hazards models, respectively. Sensitivity analyses were performed by analyzing outcomes in a peritonitis-free cohort (n = 56).

**Results:**

Dialysate IL-6 concentration significantly increased from baseline to 24 months (mean difference 19.07 pg/mL; *P* < 0.001) but was not affected by the type of PD solution received (*P* = 0.68). An increase in PSTR from baseline was associated with higher levels of IL-6 (*P* = 0.004), the use of standard solutions (*P* = 0.005) and longer PD duration (*P* < 0.001). Baseline IL-6 level was not associated with a shorter time to first peritonitis (adjusted hazard ratio 1.00, 95% CI 0.99-1.00, *P* = 0.74). Analysis of IL-6 AR as well as sensitivity analyses in a peritonitis-free cohort yielded comparable results.

**Conclusion:**

Dialysate IL-6 concentration increased with longer PD duration and was a significant, independent predictor of PSTR. The use of biocompatible PD solutions exerted no significant effect on dialysate IL-6 levels but did abrogate the increase in PSTR associated with standard PD solutions. This is the first study to examine the impact of biocompatible solutions on the utility of IL-6 in predicting PSTR and peritonitis.

## Background

The peritoneum is composed of a monolayer of mesothelial cells resting upon a thin basement membrane [1,2]. Continuous exposure to conventional peritoneal dialysis (PD) solutions contributes to progressive peritoneal injury, is an important source of local inflammation that can result in adverse functional outcomes, such as higher peritoneal solute transport rate (PSTR), a widely accepted risk factor for mortality and technique failure in PD patients [3-5].

Interleukin-6 (IL-6) is an immuno-modulatory cytokine that plays a critical role in many innate and acquired inflammatory processes [6]. It is secreted in large quantities by peritoneal mesothelial cells in response to inflammatory stimuli and modulated by exposure to PD solutions [7]. Interest in IL-6 and its role in intraperitoneal inflammation in PD patients is increasing as a result of the link from single centre studies to changes in PSTR, data that has now been corroborated by recent analysis from the large multicenter GLOBAL study [8-10]. To date there are no data on the impact of biocompatible PD solution use on IL-6 levels and its relationship to PSTR. Improvement in peritoneal membrane morphology from the use of more biocompatible solutions, characterized by neutral pH with lowered glucose degradation product (GDP), has been suggested previously [11,12]. Therefore, its use may lead to changes in the intraperitoneal inflammatory milieu with potential benefits to peritoneal membrane function and improved host defence against peritonitis. Although a number of randomized controlled trials (RCT) have reported comparable dialysate IL-6 levels from biocompatible and standard solutions use [13-16], these studies were limited by open and variable prescription, large drop out rates (>20%) [16], cross-over study designs with the risk of carry-over effect [14], risk of bias from including biocompatible solutions with variable GDP levels in the treatment group [15], and a lack of accounting for the confounding effect of peritonitis [13-16]. The utility of dialysate IL-6 as a potential predictor of adverse peritoneal membrane function has not been studied within an RCT setting.

The aims of the present study were to describe the trend in dialysate IL-6 concentration and IL-6 appearance rate (IL-6 AR) in incident PD patients, to explore the utility of dialysate IL-6 as a predictor of PSTR and peritonitis in this patient group and to evaluate the impact of neutral pH, low GDP PD solutions on these outcomes.

## Methods

### Study design

Data were obtained from the participants of the balANZ trial [17]. Detailed description of the study design and methodology has been previously published [18], as have the results of the main primary and secondary analyses [17,19,20]. The trial was registered with the Australian New Zealand Clinical Trials Registry (ACTRN12606000044527). The study protocol was approved by the ethics committees at Princess Alexandra Hospital Research Ethics Committee as well as all other participating units (see Appendix for list of hospitals). All patients provided written informed consent prior to trial participation, including consent to biomarker studies using stored samples. Incident, adult PD patients who had commenced dialysis for the first time within the preceding 90 days with both a residual measured glomerular filtration rate (GFR) ≥5 ml/min/1.73 m^2^ and a measured urine volume ≥400 ml/day at enrolment were included in the study. Pregnant or breastfeeding patients, individuals expected to die within 12 months, patients participating in trials targeting residual renal function in PD or those with a significant cancer history in the past 5 years, acute infection at enrolment, contra-indications to PD, any physical or mental disorder that appreciably hampered study protocol compliance or known or suspected allergy to trial product or related products were excluded. Of the 185 participants of the balANZ trial, 88 participants who had completed 24-month follow-up (Balance® n = 42; Stay.Safe® n = 46) with at least one PD effluent (PDE) sample stored during trial participation were included in the present investigation.

#### IL-6

PDE samples were collected at baseline, 12, and 24 month visits. PDE was initially stored in a −20°C or −80°C freezer locally, then transported frozen to a central storage facility and kept at −80°C. Samples were thawed once only during the aliquoting process prior to analysis. IL-6 was measured by an electrochemiluminescence immunoassay technique using the manufacturer’s protocols. 96-well plates measuring IL-6 were analysed on a Sector Imager 6000® (Mesoscale Discovery [MSD], Gaithusburg, MD, USA). Samples and standards were analysed in duplicates with a maximum tolerated coefficient of variation (CV) of 20%. The lower level of detection limit was 0.07 pg/mL. No inter-assay CV was determined as all samples from an individual were run at the same time to minimize between-assay variability.

#### Clinical outcomes

The clinical outcome measures were: 1) PSTR defined as 4 hour dialysate:plasma creatinine ratio (DP_cr4h_) measured during the peritoneal equilibration test (PET), and 2) episodes of PD-related peritonitis during study participation.

#### Calculations

IL-6 AR was calculated by multiplying the PDE IL-6 concentrations and drained volume, which was then divided by the dwell time and expressed as picograms per minute. To account for individual variation in baseline IL-6 concentrations and peritoneal functional capacity, the change in IL-6, IL-6 AR, and PSTR were calculated for month 12 (month 12 – baseline); and month 24 (month 24 – baseline). Baseline values of PSTR were measured at month 1.

### Statistical analyses

Results were expressed as frequencies (percentages) for categorical variables, mean ± standard deviation (SD) for continuous normally distributed variables, and median (interquartile range) for continuous non-normally distributed variables. Differences between groups on baseline characteristics were analysed by χ^2^ test for categorical data, t-test or Mann–Whitney U test for continuous data, as appropriate. The overall trend in IL-6 concentrations and IL-6 AR were analysed by fitting a multilevel linear regression model to the log-transformed IL-6 data (or IL-6 AR). Categorical time (i.e. baseline, 12, 24 months) was included as a fixed-effect and random intercepts and slopes were added to allow for repeated measurements over time. To evaluate the differences between the two treatment groups on IL-6, PD solution type (Biocompatible vs. Control) and the interaction between solution type and time were subsequently added to the model as fixed effects. If the interaction term was not statistically significant, the unconditional effects of PD solution type on IL-6 would be retained and the interaction term was removed from the final model. In addition, the baseline IL-6 or IL-6 AR were explored as potential predictors of change in their respective post-baseline levels. The IL-6 and IL-6 AR data were log transformed due to their non-normal distributions. As a sensitivity analysis, the role of loss of residual renal function (calculated by GFR at relevant month – baseline value) on IL-6 concentrations over time was explored as a time-varying covariate in the multilevel linear regression model.

To determine whether IL-6 was associated with changes in PSTR, a multilevel linear regression model was fitted. Clinically recognized risk factors that affect PSTR (such as time on PD, racial origin, age, body mass index [BMI], male gender) [21,22] and randomly assigned type of PD solution [19] were included as fixed-effects in an initial full model. Variables with statistically non-significant effects in the full model were removed from the final model. The fit of the final model was checked against the full model using the likelihood ratio test. Any significant interaction identified was added to the model. Due to the potential bias introduced by peritonitis-related increase in IL-6 and PSTR, a sensitivity analysis in a subgroup who were peritonitis-free was performed (biocompatible n = 34; control n = 22).

Time to first peritonitis was analyzed by a multivariable Cox proportional hazards model. Baseline IL-6, PD solution type, age, sex, racial origin, BMI, diabetes mellitus, PD modality (automated PD/continuous ambulatory PD) were explored as covariates. Baseline IL-6 was chosen instead of IL-6 from each visit because of the cause-and-effect relationship between IL-6 and peritonitis. Patients who experienced peritonitis prior to the first PDE collection were excluded from the analysis (n = 3). Data were analysed using the software package Stata/SE12.0 (College Station, TX). P < 0.05 was considered to represent statistically significant differences.

## Results

### Patient characteristics

The patients (biocompatible n = 42; control n = 46) were well matched for all baseline characteristics other than higher peritoneal ultrafiltration (*P* = 0.03) and lower PSTR (*P* = 0.006) at one month in the control group (Table 1). The baseline characteristics of this subgroup were comparable to the original balANZ trial cohort [17].

**Table 1:** Baseline characteristics

**Characteristic**	**Total (n=88)**	**Biocompatible (n=42)**	**Control (n=46)**	**P values**
Age (yr)	62 (51–69)	64 (51–70)	60 (51–67)	0.28
Female	39 (44.32)	18 (42.86)	21 (45.65)	0.79
Ethnicity				0.21
- Caucasian	65 (73.86)	34 (80.95)	31 (67.39)	
- ATSI	3 (3.41)	1 (2.38)	2 (4.35)	
- Asian	16 (18.18)	7 (16.67)	9 (19.57)	
- MPI	4 (4.55)	0 (0)	4 (8.69)	
Diabetes Mellitus	28 (31.82)	12 (28.57)	16 (34.78)	0.53
Primary renal disease				0.16
- Glomerulonephritis	21(23.86)	9 (21.43)	12(26.09)	
- Diabetic nephropathy	22 (25.00)	7 (16.67)	15 (32.61)	
- Hypertensive/Renovascular	15 (17.05)	7 (16.67)	8 (17.39)	
- Polycystic kidney disease	8 (9.09)	5 (11.90)	3 (6.52)	
- Reflux nephropathy	4 (4.55)	4 (9.52)	0 (0.00)	
- Other	18 (20.45)	10 (23.81)	8 (17.39)	
Body mass index (kg/m^2^)				0.54
- <20	2 (2.27)	0 (0)	2 (4.35)	
- 20-24.9	25 (28.41)	13 (30.95)	12 (26.09)	
- 25-30	32 (36.36)	16 (38.10)	16 (34.78)	
- >30	29 (32.95)	13 (30.95)	16 (34.78)	
Haemodialysis before PD	4 (4.55)	2 (4.76)	2 (4.35)	0.93
Initial PD modality				0.79
- CAPD	81 (92.05)	39 (92.86)	42 (91.30)	
- APD	7 (7.95)	3 (7.14)	4 (8.70)	
GFR (mL/min per 1.73 m^2^)	7.40 ± 2.85	7.28 ± 2.58	7.51 ± 3.09	0.72
Urine volume (mL/day)	1379.50 (1057.5-1900)	1573 (1060–2059)	1325 (1055–1810)	0.25
7.5% Icodextrin use (%)	11 (12.50)	5 (11.90)	6 (13.04)	0.87
Ultrafiltration^#^ (mL/day)	959.50 (510–1400)	800 (430–1300)	1050 (773–1500)	0.03
Normalized ultrafiltration corrected for total glucose exposure^#^	4.08 (2.21-6.04)	3.18 (1.78-4.19)	4.59 (3.17-7.45)	0.002
Total glucose exposure (g/day)	122.52 ± 32.44	122.38 ± 32.35	122.65 ± 32.90	0.97
D:P_Cr4h_^*^	0.64 ± 0.10	0.67 ± 0.11	0.61 ± 0.09	0.006
IL-6 (pg/mL)	7.22 (3.93-13.71)	7.61 (4.66-19.98)	6.42 (3.29-9.63)	0.17
IL-6 Appearance rate^^^	23.19 (13.66-59.35)	27.14 (14.12-67.27)	21.90 (13.66-35.01)	0.45

^#^Measured at month 3; ^*^Measured at month 1; ^^^IL-6 appearance rate = IL-6*effluent volume/dwell time in minutes.

APD: automated peritoneal dialysis; ATSI: Aboriginal and Torres Strait Islander; CAPD: continuous ambulatory peritoneal dialysis; D:PCr4h: dialysate:plasma creatinine ratio measured at 4 hours; GFR: glomerular filtration rate; IL-6: interleukin-6; MPI: Maori & Pacific Islander; PD: peritoneal Dialysis.

### The trend of dialysate interleukin-6 concentrations

**Figure 1:**
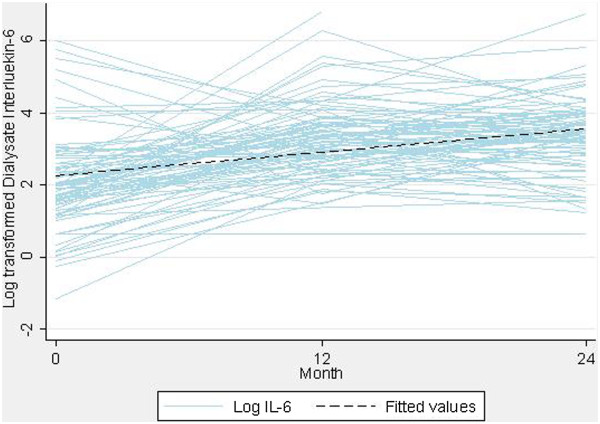
Overall trend of log-transformed dialysate Interleukin-6 concentrations over time in incident peritoneal dialysis patients.

**Figure 2:**
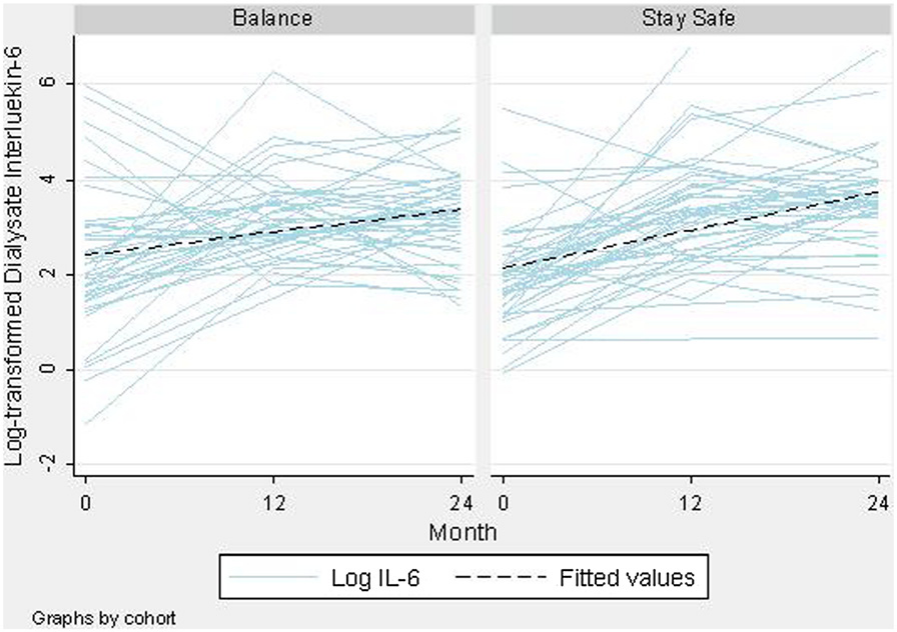
Trend of log-transformed dialysate Interleukin-6 concentrations over time by type of peritoneal dialysis solutions received (Biocompatible n = 42; Control n = 46).

There was a statistically significant increase in median IL-6 concentrations over time from 7.22 pg/mL at baseline to 31.35 pg/mL at month 24 (*P* < 0.001, Figure 1). Similar trajectories were observed for the biocompatible and control groups (*P* = 0.68; Figure 2). There was no significant interaction between the type of PD solution received and time on PD (*P* = 0.09; data not shown). PD duration remained a significant and independent predictor of IL-6 after the type of PD solutions received was added to the model (Table 2). There was no significant association between loss of residual renal function and the time trend in dialysate IL-6 concentrations (*P* = 0.67). Change in IL-6 concentration from baseline was comparable at 12 and 24 months (*P* = 0.12) but was negatively associated with baseline IL-6 levels (coefficient −0.88, *P* < 0.001; Additional file 1: Figure S1). A sensitivity analysis excluding an outlier produced comparable results (coefficient −0.88, *P* < 0.001). Similar results were obtained when analyses were repeated in the peritonitis-free cohort (Table 2) as well as when IL-6 AR (Additional file 1: Table S1) was analysed as the outcome.

**Table 2:** Multilevel linear regression to evaluate the effect of peritoneal dialysis solution type on dialysate interleukin-6 concentrations

	**All patients (n=88)**			**Peritonitis-free patients (n=56)**		
**Variable**	**Regression coefficient**	**Standard error**	**P values**	**Regression coefficient**	**Standard error**	**P values**
PD solution						
- Control	Reference	Reference	Reference	Reference	Reference	Reference
- Biocompatible	−0.07	0.17	0.68	−0.14	0.22	0.54
Time			<0.001			<0.001
- Month 0	Reference	Reference	Reference	Reference	Reference	Reference
- Month 12	1.18	0.14	<0.001	1.27	0.17	<0.001
- Month 24	1.30	0.17	<0.001	1.40	0.22	<0.001

### Interleukin-6 as predictor of peritoneal membrane function

Change in PSTR from baseline was significantly greater in those with higher levels of IL-6 (*P* = 0.004), who had received standard PD solutions (*P* = 0.005), and over longer PD duration (*P* < 0.001). The two-way interaction between PD duration and PD solution type was significant (*P* = 0.007), such that patients who received standard solutions experienced a greater increase in PSTR with longer PD duration whilst those who received biocompatible solutions maintained a relatively stable PSTR over time (Table 3; Additional file 1: Figure S2). A sensitivity analysis in the peritonitis-free cohort produced similar results (Additional file 1: Table S2) except that the effect of standard solutions on PSTR was no longer statistically significant (*P* = 0.06).

**Table 3:** Multilevel linear regression of change in peritoneal solute transport rate in incident peritoneal dialysis patients

	**Full model**			**Final model**		
**Variable**	**Coefficient**	**Standard error**	**P values**	**Coefficient**	**Standard error**	**P values**
Log_10_ IL-6	0.02	0.006	0.002	0.02	0.006	0.004
PD solution						
- Control	Reference	Reference	Reference	Reference	Reference	Reference
- Biocompatible	−0.05	0.02	0.005	−0.05	0.02	0.005
PD duration						
- 12 month	Reference	Reference	Reference	Reference	Reference	Reference
- 24 month	0.05	0.01	<0.001	0.05	0.01	<0.001
Age	0.0002	0.0007	0.74			
Male	−0.03	0.02	0.06			
BMI (kg/m2):			0.11			
- <20	Reference	Reference	Reference			
- 20-24.9	0.03	0.06	0.59			
- 25-30	0.02	0.06	0.71			
- >30	0.07	0.06	0.25			
Two-way interaction (PD duration*PD solution)^#^	−0.04	0.01	0.008	−0.04	0.01	0.007
Ethnicity			0.31			
- Caucasian	Reference	Reference	Reference			
- Asian	−0.02	0.02	0.44			
- ATSI	−0.08	0.05	0.09			
- MPI	0.01	0.04	0.74			

IL-6: Interleukin-6; PD: peritoneal dialysis; BMI: body mass index; ATSI: Aboriginal and Torres Strait Islander; MPI: Maori and Pacific Islander.

^#^Represents interaction between month 12 and use of biocompatible PD solution use; interaction term from month 24 omitted because of collinearity.

### Baseline interluekin-6 as predictor of peritonitis

Twenty-nine patients (biocompatible n = 5; control n = 24) experienced 45 episodes of peritonitis during the study. Peritonitis rates, expressed as episodes per patient-year, were 0.27 (95% confidence interval [95% CI] 0.19-0.36) overall, 0.10 (95% CI 0.04-0.20) in the biocompatible group, and 0.41 (95% CI 0.29-0.56) in the control group. The majority of patients experienced their first peritonitis in the first 12 months of the study (77.8%). Baseline median dialysate IL-6 levels in patients who experienced peritonitis against those who never experienced peritonitis were, 7.87 pg/mL and 5.75 pg/mL, respectively (*P* = 0.15). According to a multivariable Cox proportional hazards model analysis, standard solution use (biocompatible solution: adjusted hazard ratio [AHR] 0.19, 95% CI 0.07-0.50; *P* = 0.001), but not baseline IL-6 levels (AHR 1.00, 95% CI 0.99-1.00; *P* = 0.74), was associated with a significantly shorter time to first peritonitis.

## Discussion

The present investigation is the first study to examine the impact of biocompatible PD solution use on the utility of IL-6 as a predictor of higher PSTR and peritonitis in incident PD patients. A statistically significant increase in dialysate IL-6 concentrations with longer time on PD was observed whilst the type of PD solution (biocompatible vs. standard) and loss of residual renal function exerted no effect on its levels. Higher levels of dialysate IL-6 and the use of standard solutions were predictive of a greater increase in PSTR. The risk of peritonitis was associated with PD solution (biocompatible solution reduced the risk) but not baseline IL-6 levels.

An increase in dialysate IL-6 levels with a longer PD duration has been inconsistently reported by several groups [8,13,23] including the recently completed GLOBAL fluid study, which did not show any association between the duration of PD and dialysate IL-6 levels in incident and prevalent PD patients [10]. While results from the GLOBAL study were strengthened by a large number of patients (n = 959), no data were presented pertaining to a longitudinal trend in dialysate IL-6 levels nor was there a detailed examination of the impact of biocompatible PD solution use on intraperitoneal inflammatory markers. In contrast, the extension study of the Balnet trial observed a significant increase in dialysate IL-6 levels from baseline at 24 months in both biocompatible (143 ± 69.6 pg/mL vs. 57.6 ± 54.5 pg/mL; *P* < 0.001) and control groups (121 ± 69 pg/mL vs. 47 ± 31.2 pg/mL; *P* < 0.001) [13]. There was no significant difference between the two groups (*P* = 0.38). However, the sample size was small (n = 46), and the potential impact of peritonitis on dialysate IL-6 levels was not separately explored. The latter factor might have been important given the discrepant number of peritonitis episodes between biocompatible and control groups (19 vs. 6 episodes, respectively), with a significantly worse peritonitis-free survival in the biocompatible group (*P* = 0.03). The present investigation also observed a statistically significant increase in dialysate IL-6 levels with longer PD duration with a lack of observed difference between biocompatible and standard types of PD solution. The robustness of the findings was confirmed by a sensitivity analysis performed in the peritonitis-free cohort. Nevertheless, there is conflicting information about the effect of biocompatible PD solutions on dialysate IL-6 levels. For instance, the Bicarbonate/Lactate Study group (n = 92) reported a significant decrease in dialysate IL-6 levels at six months (*P* = 0.01) in the patients who received biocompatible solutions [24]. Although the difference in results might have stemmed from examining the biocompatible solution that contained bicarbonate-buffer, another study (n = 55) examining bicarbonate-buffer based solution reported comparable dialysate IL-6 levels between biocompatible and standard solution groups [14]. Therefore, the true effect of biocompatible solutions on the level of intraperitoneal inflammation, defined by IL-6 concentrations, remains uncertain. Furthermore, the rate of loss of residual renal function exerted no significant impact on dialysate IL-6 levels. These results are supported by findings from the GLOBAL study where urine volume had no significant impact on dialysate IL-6 levels in incident PD patients (*P* = 0.2) [10].

Another important observation from the current investigation was the significant role of an individual’s baseline IL-6 levels in predicting future levels. Patients with low baseline IL-6 levels yielded the greatest increase in dialysate IL-6 concentrations over time, whereas the opposing relationship held true for those with higher baseline IL-6 levels (Additional file 1: Figure S1). This highlights the importance of performing appropriate statistical analyses to accommodate intra-individual correlation and inter-individual variation, as adopted in the present study.

Similarly, due to the presence of inter-individual variation in PSTR levels, a change in PSTR from baseline for each individual was examined. Significant associations between increasing PSTR and higher IL-6 levels, the use of standard PD solutions and longer PD duration were observed. These results are in keeping with the findings from the GLOBAL study [10]. An association between a greater increment in IL-6 with increasing PSTR (r = 0.306, *P* = 0.002) was previously reported in a prospective 12-month longitudinal observational study (n = 187) in incident PD patients [23]. This study allowed for patients to receive either biocompatible or standard solutions, and observed an increase in PSTR in patients receiving standard solutions over time, unlike those who were treated using biocompatible solutions who maintained a stable PSTR. However, whether PD solution type and/or dialysate IL-6 levels had differing or independent effects on PSTR was not examined. Furthermore, the choice of therapy (biocompatible vs. standard) was at the discretion of each patient’s treating physician, thereby introducing the risk of selection bias. In contrast, the cohorts from the present study were participants of a RCT with well-balanced baseline characteristics, at low risk of selection bias and with longer follow-up duration (24 months). In addition, when PSTR was re-examined in those free of peritonitis, a consistent association with dialysate IL-6 levels was observed. These associations are certainly biologically plausible as higher PSTRs are considered a functional consequence of morphological alterations at the level of the peritoneal membrane (i.e. peritoneal membrane injury), which are likely to be a result of or lead to more marked intraperitoneal inflammation.

Progressive and cumulative peritoneal injury, mediated by repeated exposures to PD solutions [2], might not only manifest with changes in the inflammatory milieu but also increase the risk of developing further problems (e.g. peritonitis) as the balance of peritoneal homeostasis and host defence are altered [25]. In this respect the IL-6 and associated signaling cascades play a key role in controlling host defence and leukocyte trafficking during infection [6,26]. In fact, a retrospective observational study of incident PD patients (n = 31) receiving standard solutions reported higher baseline dialysate IL-6 concentrations in patients who developed peritonitis (58.4 ± 12.6 pg/mL) compared to those who remained peritonitis-free (20.3 ± 8.7 pg/mL, *P* = 0.07) [8]. The difference however did not reach statistical significance, and the study was limited by its small sample size and single-centre design. Similar to their findings, a median dialysate IL-6 concentration was greater in those who experienced peritonitis in the present study, but was not statistically significant when compared to those who did not develop peritonitis. Furthermore, only the use of standard PD solutions, and not baseline IL-6, was predictive of shorter time to first peritonitis. These findings support the results from pre-clinical trials where the use of biocompatible solutions has been associated with an improvement in host cell defence [27]. The mechanisms contributing to the beneficial effect of biocompatible solutions are clearly complex but its ‘protective effect’ may extend beyond improved preservation of the peritoneal membrane. These beliefs have been challenged by findings from a recent observational study that reported a shorter time to first peritonitis with the use of biocompatible solutions [28]. However, this study has several limitations. These include a risk of indication bias with residual confounding, relatively low usage of biocompatible solutions in the cohort suggestive of underlying selection bias, and classifying biocompatible solutions from different manufacturers with varying GDP content in the biocompatible group which could have compromised the beneficial effect from the use of solutions that are truly low in their GDP content. On the other hand, although the balANZ trial was able to clearly demonstrate a lowered risk of peritonitis with the use of biocompatible solutions, it was one of the secondary outcomes measured during the trial [17,20]. Therefore, in order to evaluate the true effect of biocompatible solutions on peritonitis risk, a well designed, multicenter, multinational RCT adequately powered to examine peritonitis as a primary outcome is needed.

The present study is strengthened by its long follow-up duration in comprehensively described participants of an RCT. It is one of the largest longitudinal studies to date exploring the utility of IL-6 in predicting important patient-level clinical outcomes and the influence of biocompatible PD solution use. Also, given the cause-and-effect relationship between peritonitis and IL-6 levels, a sensitivity analysis including only peritonitis-free patients was performed to confirm the robustness of the findings.

However, the conclusions that can be drawn from the study are challenged by several limitations. First, the balANZ trial was a RCT with primary outcome measure of residual renal function decline, rather than a biomarker study. Therefore, the study design did not account for potential biological and pre-analytical sources of variations of IL-6, which could have affected the measured results. Second, the baseline PSTRs were not true baseline values as they were obtained at one month after study commencement. Thirdly, by selecting patients who had completed the 24-month follow-up, this might have introduced the risk of selection bias. Fourthly, some of the risk factors associated with a shorter time to peritonitis, such as educational level, [29] were not available and could not be adjusted for in the relevant analyses. And lastly, although it is one of the largest studies to date, the size is relatively small and may have increased the chance of Type II statistical error especially when comparing the outcomes between biocompatible and control groups.

## Conclusions

In conclusion, dialysate IL-6 concentration increased with longer time on PD and was an independent and significant predictor of increasing PSTR. The type of PD solution did not influence dialysate IL-6 concentration levels. Standard PD solution use, but not baseline IL-6 levels, was predictive of shorter time to first peritonitis. Future studies should aim to identify the causes of higher dialysate IL-6 levels and to validate the use of IL-6 as a potential monitoring tool with an aim to assist in risk stratifying PD patients at risk of increasing PSTR.

## Appendix

### Collaborators (balANZ Investigators)

Australian Centres: G Rangan, L Liew, Blacktown Hospital, Sydney (NSW); H Kulkarni, U Steinwandel, Fremantle Hospital, Fremantle (WA); B Jones, L Garvey, John Hunter Hospital, Newcastle (NSW); M G Suranyi, M Gilbert, Liverpool Hospital, Sydney (NSW); F G Brown, I Abraham, J Nandkumar Monash Medical Centre, Melbourne (VIC); A Coburn, V Bali, Princess Alexandra Hospital, Brisbane (QLD); S McDonald, S Frasca, M Hockley, C Russ, The Queen Elizabeth Hospital, Adelaide (SA); T J Elias, K Bannister, M Hockley, K Pirone, Royal Adelaide Hospital (SA); D Ranganathan, L Williams, Royal Brisbane Hospital, Brisbane (QLD); K Warr, G Smith, Perth (WA); N Boudville, S Pellicano, Sir Charles Gairdner Hospital, Perth (WA); R Langham, E O’Flaherty, St Vincents Hospital, Melbourne (VIC).

New Zealand Centres: J Schollum, L Reed, L Anderson Dunedin Hospital, Dunedin; D Voss, B Jagannathan, P Nicholls Middlemore Hospital, Auckland.

Singapore Centres- M WY Foo, CK Tam, Singapore General Hospital, Singapore; R Lee, Tang Tock Seng Hospital, Changi General Hospital, Singapore; SH Tan Kidney and Medical Clinic, Gleneagles Medical Centre, Singapore.

## Abbreviations

BMI: Body mass index; CV: Coefficient of variation; GDP: Glucose degradation product; GFR: Glomerular filtration rate; IL-6: Interleukin-6; IL-6 AR: Interleukin-6 appearance rate; PD: Peritoneal dialysis; PDE: Peritoneal dialysis effluent; PSTR: Peritoneal solute transport rate; RCT: Randomized controlled trial.

## Competing interests

David Johnson is a consultant for Baxter Healthcare Pty Ltd and has previously received research funds from this company. He has also received speakers’ honoraria and research grants from Fresenius Medical Care. He has previously been a consultant to Gambro Pty Ltd. He is an International Society of Peritoneal Dialysis Councillor and is a current recipient of a Queensland Government Health Research Fellowship. Yeoungjee Cho is a current recipient of Australian Postgraduate Award and is a recipient of 2012 Jacquot Research Entry Scholarship. Carmel Hawley has received research grants from Baxter Healthcare Pty Ltd and Gambro Pty Ltd, and has been a consultant to Fresenius Medical care. Margaret Clarke is an employee of Fresenius Medical Care. Remaining authors declare no conflicts of interest.

## Authors’ contributions

The study was conceived, designed, and supervised by authors YC, DWJ, CH, NT, DV (non-Fresenius employee). MC was involved in the acquisition of the data. YC wrote the first draft of the manuscript, subsequent drafts were reviewed and approved by YC, DWJ, DV, CH, EP, MC, and NT.

## Pre-publication history

The pre-publication history for this paper can be accessed here:

http://www.biomedcentral.com/1471-2369/15/8/prepub

## Supplementary Material

Additional file 1: Table S1 Multilevel linear regression to evaluate the effect of peritoneal dialysis solution type on dialysate interleukin-6 appearance rate. **Table S2.** Multilevel linear regression of change in peritoneal solute transport rate in peritonitis-free incident peritoneal dialysis patients. **Figure S1.** Scatter plot depicting the relationship between baseline interleukin-6 concentrations and change in interleukin-6 from baseline at a) Month 12 and b) Month 24. **Figure S2.** Trend in change in peritoneal solute transport rate (defined using 4-hour dialysate:plasma creatinine) from baseline value according to the type of peritoneal dialysis solutions received. Click here for file
