# Supplementary material for: Dialysate interleukin-6 predicts increasing peritoneal solute transport rate in incident peritoneal dialysis patients
Source: BMC Nephrol. 2014 Jan 10;15:8. doi: 10.1186/1471-2369-15-8 (PMC3893539; doi:10.1186/1471-2369-15-8)
Supplement: Additional file 1: Table S1 — Multilevel linear regression to evaluate the effect of peritoneal dialysis solution type on dialysate interleukin-6 appearance rate. Table S2. Multilevel linear regression of change in peritoneal solute transport rate in peritonitis-free incident peritoneal dialysis patients. Figure S1. Scatter plot depicting the relationship between baseline interleukin-6 concentrations and change in interleukin-6 from baseline at a) Month 12 and b) Month 24. Figure S2. Trend in change in peritoneal solute transport rate (defined using 4-hour dialysate:plasma creatinine) from baseline value according to the type of peritoneal dialysis solutions received. [file 1471-2369-15-8-S1.doc]

**Additional file 1: Table S1.** Multilevel linear regression to evaluate the effect of peritoneal dialysis solution type on dialysate interleukin-6 appearance rate.

|  | **All patients (n=88)** | | | **Peritonitis-free patients (n=56)** | | |
| --- | --- | --- | --- | --- | --- | --- |
| **Variable** | **Regression coefficient** | **Standard error** | **P values** | **Regression coefficient** | **Standard error** | **P values** |
| PD solution  - Control  -Biocompatible | Reference  -0.14 | Reference  0.17 | Reference  0.42 | Reference  -0.21 | Reference  0.23 | Reference  0.35 |
| Time   - Month 0 - Month 12 - Month 24 | Reference  1.28  1.38 | Reference  0.14  0.17 | <0.001  Reference  <0.001  <0.001 | Reference  1.33  1.46 | Reference  0.17  0.22 | <0.001  Reference  <0.001  <0.001 |

**Additional file 1: Table S2.** Multilevel linear regression of change in peritoneal solute transport rate in peritonitis-free incident peritoneal dialysis patients.

|  | **Full Model** | | | **Final Model** | | |
| --- | --- | --- | --- | --- | --- | --- |
| **Variable** | **Coefficient** | **Standard Error** | **P values** | **Coefficient** | **Standard Error** | **P values** |
| Log_10_ IL-6 | 0.02 | 0.008 | 0.03 | 0.01 | 0.007 | 0.04 |
| PD solution  - control  - biocompatible | Reference  -0.04 | Reference  0.02 | Reference  0.08 | Reference  -0.05 | Reference  0.02 | Reference  0.06 |
| PD duration   - 12 month - 24 month | Reference  0.05 | Reference  0.01 | Reference  0.001 | Reference  0.05 | Reference  0.01 | Reference  0.001 |
| Age | -0.0009 | 0.0009 | 0.32 |  |  |  |
| Male | -0.02 | 0.02 | 0.35 |  |  |  |
| BMI (kg/m2):   - 20-24.9^ - 25-30 - >30 | Reference  -0.02  0.05 | Reference  0.03  0.03 | 0.04  Reference  0.45  0.10 | Reference  -0.01  0.04 | Reference  0.03  0.03 | 0.09  Reference  0.62  0.13 |
| Two-way interaction (PD duration*PD solution)^#^ | -0.04 | 0.02 | 0.02 | -0.04 | 0.02 | 0.02 |
| Ethnicity   - Caucasian - Asian - ATSI - MPI | Reference  0.006  -0.07  -0.008 | Reference  0.03  0.06  0.09 | 0.69  Reference  0.86  0.24  0.93 |  |  |  |

IL-6: Interleukin-6; PD: peritoneal dialysis; BMI: body mass index; ATSI: Aboriginal and Torres Strait Islander; MPI: Maori and Pacific Islander

^No patient with BMI <20kg/m2 in the peritonitis-free cohort. ^#^Represents interaction between month 12 and use of biocompatible PD solution use; interaction term from month 24 omitted because of collinearity.

**Additional file 1: Figure S1.** Scatter plot depicting the relationship between baseline interleukin-6 concentrations and change in interleukin-6 from baseline at a) Month 12 and b) Month 24.

**A.**

**B.**

**Additional file 1: Figure S2.** Trend in change in peritoneal solute transport rate (defined using 4-hour dialysate:plasma creatinine) from baseline value according to the type of peritoneal dialysis solutions received.
